# Polymorphism in the Promoter Region of the *IL18* Gene and the Association With Severity on Paracoccidioidomycosis

**DOI:** 10.3389/fimmu.2020.542210

**Published:** 2020-10-01

**Authors:** Paula Keiko Sato, Felipe Delatorre Busser, Flávia Mendes da Cunha Carvalho, Alexandra Gomes dos Santos, Aya Sadahiro, Constancia Lima Diogo, Adriana Satie Gonçalves Kono, Maria Luiza Moretti, Olinda do Carmo Luiz, Maria Aparecida Shikanai-Yasuda

**Affiliations:** ^1^Laboratory of Medical Investigation in Immunology (LIM48), Faculdade de Medicina, Hospital das Clínicas, University of São Paulo, São Paulo, Brazil; ^2^Institute of Tropical Medicine, Faculdade de Medicina, University of São Paulo, São Paulo, Brazil; ^3^Department of Infectious and Parasitic Diseases, Faculdade de Medicina, University of São Paulo, São Paulo, Brazil; ^4^Department of Parasitology, Instituto de Ciências Biológicas, Federal University of Amazonas, Manaus, Brazil; ^5^Infectious Diseases Division, Hospital das Clínicas, University of São Paulo, São Paulo, Brazil; ^6^Faculdade de Ciências Médicas, Hospital das Clínicas, State University of Campinas, Campinas, Brazil; ^7^Department of Preventive Medicine, Faculdade de Medicina, University of São Paulo, São Paulo, Brazil

**Keywords:** paracoccidioidomycosis, *Paracoccidioides* spp., single nucleotide polymophism, IL18, IL12A, IFNGR1, clinical forms of paracoccidioidomycosis

## Abstract

Paracoccidioidomycosis (PCM) is an important endemic, systemic disease in Latin America caused by *Paracoccidioides* spp. This mycosis has been associated with high morbidity and sequels, and its clinical manifestations depend on the virulence of the infecting strain, the degree and type of immune response, infected tissues, and intrinsic characteristics of the host. The T helper(Th)1 and Th17/Th22 cells are related to resistance and control of infection, and a Th2/Th9 response is associated with disease susceptibility. In this study, we focused on interleukin(IL)-12p35 (*IL12A*), IL-18 (*IL18*), and IFN-γ receptor 1 (*IFNGR1*) genetic polymorphisms because their respective roles have been described in human PCM. Real-time PCR was employed to analyze *IL12A*-504 G/T (rs2243115), *IL18*-607 C/A (rs1946518), and *IFNGR1*-611 A/G (rs1327474) single nucleotide polymorphisms (SNP). One hundred forty-nine patients with the acute form (AF), multifocal chronic (MC), or unifocal chronic (UC) forms of PCM and 110 non-PCM individuals as a control group were included. In the unconditional logistic regression analysis adjusted by ethnicity and sex, we observed a high risk of the *IL18*-607 *A*-allele for both AF [*p* = 0.015; OR = 3.10 (95% CI: 1.24–7.77)] and MC groups [*p* = 0.023; OR = 2.61 (95% CI: 1.14–5.96)] when compared with UC. The *IL18*-607 *A*-allele associated risk for the AF and MC groups as well as the protective role of the *C*-allele in UC are possibly linked to higher levels of IL-18 at different periods of the course of the disease. Therefore, a novel role of *IL18*-607 C/A SNP is shown in the present study, highlighting its importance in the outcome of PCM.

## Introduction

Paracoccidioidomycosis (PCM) is one of the main endemic, systemic mycoses in Latin America, and it is caused by the thermally dimorphic fungi of the *Paracoccidioides brasiliensis* (*P. brasiliensis*) complex (*Paracoccidioides* spp.) and *Paracoccidioides lutzii*. PCM is associated with high morbidity and sequels; however, because it is not a compulsorily notified disease in Brazil, the actual data are based on reports of epidemiological surveys, case series, hospitalization, and mortality data (1). It is endemic in the southeast, central west, and south regions of Brazil and estimated at 0.71–2.70/100,000 inhabitants/year (2). Epidemic areas have been reported in the western Brazilian areas with a mean incidence of 9.4/100,000 and peaks of 37–39/100,000 inhabitants (3).

The inhalation of conidia of *Paracoccidioides* spp. can result in infection without symptoms, acute or chronic disease, or in sequelae. The clinical manifestations depend on the virulence of the infecting strain, the degree and type of immune response, infected tissues, and intrinsic characteristics of the host (4).

Some components of the innate immunity, such as neutrophils, dendritic cells, toll-like receptors, dectin-1, myeloid differentiation primary response 88 (MyD88), and NOD-like receptor P3 (NLRP3) inflammasome, have been evaluated in both experimental and human PCM (5–10). However, it is the adaptive response to *P. brasiliensis* that has been extensively studied with a well-established murine model and characterization of clinical forms of PCM by T helper (Th) responses and antibodies (11).

The acute form of PCM shows a mixed Th2/Th9 response: increased levels of IL-4, IL-5, IL-9, IL-10, TGF-β, and IL-27; low production of IFN-γ and TNF-α; and high levels of specific IgG4 and IgE antibodies. On the other hand, the chronic form presents a Th17/Th22 profile with high production of IL-17 and IL-22 and also secreting Th1-type cytokines, such as IFN-γ, TNF-α, and IL-2 as well as variable levels of IL-10 and IL-4 and increased levels of specific IgG1 antibodies (12, 13). Therefore, the Th2/Th9 responses can be associated with susceptibility to PCM, and the presence of Th1 and Th17/Th22 cells can contribute to more mild clinical manifestations with the axis IFN-γ/IL-12 directly associated with protection and control of the infection (11–16).

The high-affinity binding of IL-12 to its receptor results in the differentiation of naïve CD4 T cells into Th1 lymphocytes, which are the major producers of IFN-γ, alongside NK cells. In the presence of IL-12, IL-18 also stimulates the production of IFN-γ, inducing a Th1-mediated immune response; in the absence of IL-12, IL-18 can stimulate a Th2 response (17, 18).

Higher levels of IL-18 and sTNF-RII are described in the acute form of PCM when compared with the chronic form and controls, and IL-12 is also higher in patients than in controls (19). The same group reports higher levels of IL-18 during treatment and lower levels after antifungal treatment (20). As the disease is more severe in the acute form with high IL-18 levels, the authors suggest that this cytokine could be a useful marker of PCM severity.

Previous studies on murine PCM have shown that IL-12 and IL-18 secretions are associated with innate immunity factors. MyD88-deficient mice show a more severe disease after 8 weeks of infection with low levels of IL-12, and a protective role in murine pulmonary PCM was shown in the NLRP3 inflammasome, associated with IL-1β and IL-18 secretion and expansion of Th1 and Th17 cells and suppressive control of T-reg cells (7, 21).

The mechanisms controlling these effects are unclear, but reports on genetic background in both human and experimental disease have been shedding light on this matter. For instance, an autosomal dominant gene has been associated with resistance in murine PCM (22), and in human disease, reports show the influence of the human leukocyte antigen (HLA) in both susceptibility and outcomes. The HLA class I, HLA-B13, was found in a higher proportion in PCM patients compared to controls as well as a higher frequency of HLA-A9 in patients with the progressive pulmonary form (23). In parallel, increased risk of PCM development was associated with the presence of HLA-B40, which was found more in patients than in controls (24, 25). In 2011, the class II-HLA-DRB1^*^11 allele was reported in a higher frequency in patients with the more benign clinical presentation of this disease, the unifocal chronic (UC) form (26). Moreover, PCM patients were shown to have a higher proportion of the non-expressed C4B allele, C4B^*^Q0, of deficient C4 isotypes, suggesting to the authors a possible influence of different C4 isotype and allotype frequencies in the course of infection (27).

In addition, an enzyme phenotype (GLO-1 phenotype of glyoxalase I) was associated with PCM infection represented by a positive intradermic reaction, and a possible relationship with HLA antigens deserves further discussion because there is a close linkage between the GLO phenotype and HLA (28).

Our group and others have been studying single nucleotide polymorphisms (SNPs) and different mutations on cytokines and receptor genes. For instance, the inherited mutation Leu77Phe on the IL-12 receptor β1 subunit (IL-12Rβ1) gene (*IL12RB1*) associated with its loss of function and complete deficiency, resulting in a severe, acute form of PCM (29). Moreover, in Brazilian patients with PCM, reports of polymorphisms on IL-4 and IL-12Rβ1 genes have shown the relevance of *IL4*-590 C/T and *IL12RB1* 641 A/G SNPs in association with infection or clinical forms, contributing to a better understanding of the immunopathogenesis of this disease (30, 31).

Polymorphisms on the IFN-γ gene have also been described in infectious diseases, such as toxoplasmosis, tegumentar leishmaniasis, and PCM, and no association between alleles or genotypes and these diseases was observed (31–35). Furthermore, it is also necessary to consider that the expression of the IFN-γ receptor could be interfering in the axis IL-12/IFN-γ because its deficiency was already described in the more disseminated forms of histoplasmosis, coccidioidomycosis, mycobacteriosis, and disseminated BCG infection (36–39). This deficient expression could be associated with a mutation in the IFN-γ receptor 1 gene, such as the *IFNGR1*-611 A/G SNP, which has been associated with strong promoter activity and with decreased risk of pulmonary tuberculosis (40, 41).

IL-12p70, the bioactive form of IL-12, is a heterodimer of two subunits: p35 (encoded by the *IL12A* gene) and p40 (encoded by the *IL12B* gene). No association was found between *IL12B* +1188 A/C SNP and PCM (31). SNPs in the *IL12A* gene, on the other hand, have not yet been explored in this mycosis.

Studies show an association of *IL18*-607 C/A SNP (rs1946518) with protection or risk in several infections (42–45). However, as with the *IL12A*-504 G/T (rs2243115) and the *IFNGR1*-611 A/G (rs1327474) SNPs, there are still no studies associating this genetic polymorphism with systemic fungal infections.

Considering the interaction between IL-18, IL-12, and IFN-γ and the genetic aspects possibly involved in susceptibility to PCM and the lack of studies on this disease, the present study aimed to analyze the *IL18*-607 C/A (rs1946518), *IL12A*-504 G/T (rs2243115), and *IFNGR1*-611 A/G (rs1327474) SNPs, for the first time to our knowledge, in a cohort of Brazilian patients with PCM, also presenting a brief review of studies on these SNPs in infectious diseases.

## Materials and Methods

### Subjects

A total of 149 patients with PCM from the General Infirmary and Systemic Mycosis Outpatient Clinic from the Infectious Diseases Division (Hospital das Clínicas, Faculdade de Medicina, University of São Paulo—HCFMUSP, São Paulo, SP, Brazil) were included in the study (**Table 2**). Thirty-nine patients had the acute form of PCM (AF) and 110 had the chronic form (CF); 93 had the multifocal chronic form (MC) and 17 had the UC form. This classification was performed according to Franco et al. (46). UC includes only patients with mild, restricted disease to the skin, mucosae, or lymph nodes without lung or cerebral involvement. The control group (CO) included 110 non-PCM subjects. The study protocol was approved by the ethics committee of HCFMUSP (CAPPesq 10273; Plataforma Brasil 123334/2013), and all subjects gave written informed consent in accordance with the Declaration of Helsinki.

The inclusion criteria were (a) patients with PCM: identification of *Paracoccidioides* spp. by mycological and/or histopathological examination and/or presence of anti-*P. brasiliensis* serum antibodies (titers ≥32 on counterimmunoelectrophoresis test) at the moment of enrollment or proven in the past; and (b) CO: individuals considered healthy without a previous history of the disease, not sensitized in lymphoproliferation assays against the 43-kDa glycoprotein of *P. brasiliensis*, and absence of serum anti-*P. brasiliensis* antibodies (by immunodiffusion test). Subjects with comorbidities, such as neoplasia and other acute or chronic systemic infectious diseases, were excluded.

### DNA Extraction

Genomic DNA was obtained from peripheral blood leukocytes by the salt precipitation method (DTAB/CTAB; dodecyl trimethyl ammonium bromide/cetyltrimethyl ammonium bromide, both from Sigma-Aldrich, Merck, St. Louis, MO, USA) as previously described (47). The concentration and purity of the extracted DNA were evaluated by a UV spectrophotometer (Nanodrop LITE, Thermo Fisher Scientific, Carlsbad, CA, USA).

### Detection of SNPs

The SNPs in the IL-12p35 (*IL12A*-504 G/T, rs2243115), IL-18 (*IL18*-607 C/A, rs1946518), and IFN-γ receptor 1 (*IFNGR1*-611 A/G, rs1327474) genes were investigated by real-time PCR using specific oligonucleotides and probes labeled with VIC (wild allele) or FAM (mutated allele) fluorochromes and TaqMan™ Genotyping Master Mix (all from Molecular Probes, Thermo Fisher Scientific, Carlsbad CA, USA). The assays and results were performed and analyzed with the StepOne Plus Real Time PCR System and software (Applied Biosystems, Thermo Fisher Scientific, Foster City, CA, USA).

### Statistical Analysis

The deviations from the Hardy–Weinberg equilibrium and the distribution of genotypic and allelic frequencies of the SNPs on *IL12A, IL18*, and *IFNGR1* genes in the studied population were evaluated by Pearson's χ^2^ test. To estimate the risk of patients with AF, MC, and UC PCM associated with genotypes and alleles, odds ratios (ORs) and 95% confidence intervals (95% CIs) were calculated as approximations of relative risk using unconditional logistic regression analysis. For ordered variables, tests for linear trend were done by categorizing the exposure variables and entering the scores as continuous. To verify the strength of association between the final events, we performed univariate and multivariate logistic analyses adjusted by ethnicity and sex with the STATA 14.0 software (StataCorp, College Station, TX, USA), and *p*-values ≤ 0.05 were considered statistically significant.

## Results

Considering the plausible role of genetic background in human PCM, we summarize all previously reported SNPs or mutations on immune-related genes and their main results on Brazilian patients in Table 1. Eight SNPs or mutations on DC-SIGN, HLA, IL-4, IL-10, IL-12Rβ1, and Vitamin D receptor genes were found to have an association with risk, outcome, or clinical forms of PCM. As the SNPs of this study had not yet been evaluated on PCM patients, we also compiled previous reports on the *IL18* (-607 C/A, rs1946518 and -137 G/C, rs187238), *IL12A* (-504 G/T, rs2243115), and *IFNGR1* (-611 A/G, rs13277474) SNPs and their association with diverse infectious diseases (Supplementary Tables 1–3, respectively).

**Table 1:** Polymorphisms on immune-related genes in Brazilian patients with PCM.

**Structure (gene)**	**Mutation (rs)**	**Main results**	**References**
CTLA-4 (*CTLA4*)	−318 C/T and +49 A/G^a^	• No differences between patients and controls	(48)
DC-SIGN (*DCSIGN*)	rs4804803	• Genotype *DCSIGN*-*GG* on patients with oral PCM (*p* = 0.032)	(49)
FCγ-RIIa (*FCGR2A*)	rs1801274	• No differences between patients and controls	(49)
HLA (*HLA*)	HLA-DRB1 and HLA-DQB1 alleles^a^	• HLA-DRB1*11 allele associated with UC form of PCM (*p* = 0.039)	(26)
IFN-γ (*IFNG*)	+874 T/A^a^	• No differences between patients and controls	(35)
	+874 T/A (rs2430561)	• No differences between patients and controls	(31)
		• No differences between clinical forms of PCM	
IL-4 (*IL4*)	−590 T/C^a^	• Patients with *C*-allele produce more IL-4 than those with *T*-allele (*p* < 0.05)	(35)
		• No differences between patients and controls	(30)
	Intron-3 microsatellite *RP1*/*RP2*^a^	• Genotypes *RP2*/*RP2* on patients and *RP1*/*RP1* on controls (with low IL-4 expression): *p* = 0.0042	(30)
IL-10 (*IL10*)	−1082 G/A^a^	• Genotypes *IL10-GG* on patients and *IL10*-*AA* on controls (*p* = 0.0218)	(34)
IL-12p40 (*IL12B*)	+1188 A/C (rs3212227)	• No differences between patients and controls	(31)
		• No differences between clinical forms of PCM	
IL-12Rβ1 (*IL12RB1*)	230 T/C Leu77Phe^a^	• Inherited IL-12Rβ1 deficiency leading to acute form of PCM	(29)
	641 A/G (rs11575834)	• No differences between patients and controls	(31)
		• Male patients: Genotype *IL12RB1*-*AA* on MC form and *IL12RB1*-*AG* on UC form (*p* = 0.048)	
JAK1	rs11208534	• No differences between patients and controls	(49)
TNF-α (*TNFA*)	−308 G/A^a^	• No differences between patients and controls	(34)
	rs1800629	• No differences between patients and controls	(49)
Vitamin D Receptor (*VDR*)	rs7975232	• Genotype *VDR*-*CC* (*p* < 0.001) and *C*-allele (*p* = 0.027) on patients	(49)

a *rs not informed or not applicable*.

This study evaluated the *IL18*-607 C/A, *IL12A*-504 G/T, and *IFNGR1*-611 A/G SNPs in 149 patients with the AF and CF of PCM and 110 control individuals (CO). The CF group is subclassified as MC and UC forms. There were no statistically significant differences between cases and controls in the distribution of ethnic groups or sex (data not shown). Regarding the different groups of patients (AF, MC, and UC), no differences in ethnic distribution were found (*p* = 0.476). However, in the univariate analysis of sex distribution, there was a significant difference between these groups with a male majority on a ratio of 17.6 in the MC group (Table 2).

**Table 2:** Distribution (*n*), sex (M = Male; F = Female) and ethnicity (W = White; B = Black) ratio, and *p*-values among the groups of patients with acute, multifocal chronic and unifocal chronic forms of PCM.

**Clinical forms of PCM**	***n***	**Sex ratio (M/F)**	***p***	**Ethnicity ratio (W/B)**	***p***
Acute	39	1.8 (25/14)	0.000	1.2 (12/10)	0.476
Multifocal chronic	93	17.6 (88/5)		2.1 (57/27)	
Unifocal chronic	17	2.4 (12/5)		2.5 (10/4)	

Genotypic frequencies for the three evaluated SNPs were in Hardy–Weinberg equilibrium (data not shown). The distribution of genotypic and allelic frequencies of *IL12A*-504 G/T, *IL18*-607 C/A, and *IFNGR1*-611 A/G SNPs among patients and controls and among AF and CF groups is shown in Table 3. We found no statistical significances in the univariate analysis of genotypic frequencies in the codominant, dominant, and recessive models nor in the distribution and frequencies of alleles.

**Table 3:** Genotypic and allelic distributions and frequencies of *IL12A*-504 G/T, *IL18*-607 C/A, and *IFNGR1*-611 A/G SNPs, values of Odds Ratio (OR) and 95% Confidence Interval (95% CI) among the groups of Controls (*n* = 110) and Patients (*n* = 149), and among the groups of patients with Acute (*n* = 39) and Chronic (Multifocal + Unifocal) forms of PCM (*n* = 110).

**SNP**	**Genotypes**	**Alleles**	**Groups—*****n*** **(%)**			**Clinical forms of PCM—*****n*** **(%)**		
			**Controls**	**Patients**	**OR (95% CI)**	**Acute**	**Chronic**	**OR (95% CI)**
*IL12A*-564 G/T	GG		2 (1.8)	2 (1.3)	1.0	1 (2.6)	1 (0.8)	1.0
	GT		28 (25.5)	40 (26.9)	1.43 (0.19–10.76)	13 (33.4)	27 (24.6)	0.48 (0.03–8.32)
	TT		80 (72.7)	107 (71.8)	1.34 (0.18–9.7)	25 (64.0)	82 (74.6)	0.3 (0.02–5.05)
		G	32 (14.5)	44 (14.8)	1.0	15 (19.2)	29 (13.2)	1.0
		T	188 (85.5)	254 (85.2)	0.98 (0.6–1.61)	63 (80.8)	191 (86.8)	0.63 (0.32–1.26)
*IL18*-607 C/A	CC		29 (26.4)	44 (29.5)	1.0	10 (25.7)	34 (30.9)	1.0
	CA		60 (54.5)	68 (45.6)	0.75 (0.42–1.34)	16 (41.0)	52 (47.3)	1.05 (0.43–2.58)
	AA		21 (19.1)	37 (24.8)	1.16 (0.57–2.37)	13 (33.3)	24 (21.8)	1.84 (0.7–4.89)
		C	118 (53.6)	156 (52.3)	1.0	36 (46.2)	120 (54.5)	1.0
		A	102 (46.4)	142 (47.7)	1.05 (0.74–1.49)	42 (53.8)	100 (45.5)	1.4 (0.83–2.35)
*IFNGR1*-611 A/G	AA		14 (12.8)	21 (14.1)	1.0	4 (10.3)	17 (15.5)	1.0
	AG		47 (43.1)	66 (44.3)	0.93 (0.43–2.03)	19 (48.7)	47 (42.7)	0.58 (0.17–1.96)
	GG		48 (44.0)	62 (41.6)	0.86 (0.4–1.87)	16 (41.0)	46 (41.8)	0.67 (0.2–2.3)
		A	75 (34.4)	108 (36.2)	1.0	27 (34.6)	81 (36.8)	1.0
		G	143 (65.6)	190 (63.8)	0.92 (0.64–1.33)	51 (65.4)	139 (63.2)	1.1 (0.64–1.89)

In Table 4, we show the distribution of genotypic and allelic frequencies of *IL12A*-504 G/T, *IL18*-607 C/A, and *IFNGR1*-611 A/G SNPs among the groups of patients with the different clinical forms of PCM, analyzed by unconditional logistic regression with adjustments for ethnicity and sex. The comparison between AF, MC, and UC genotypic distribution did not result in a statistical difference.

**Table 4:** Genotypic and allelic distributions and frequencies of *IL12A*-504 G/T, *IL18*-607 C/A, and *IFNGR1*-611 A/G SNPs, values of Odds Ratio (OR) and 95% Confidence Interval (95% CI) adjusted for sex and race by unconditional logistic regression analysis among the groups of patients with Acute (AF, *n* = 39), Multifocal Chronic (MC, *n* = 93) and Unifocal Chronic (UC, *n* = 17) forms of PCM.

**SNP**	**Model**	**Genotypes**	**Alleles**	**Clinical Forms of PCM—*****n*** **(%)**			**AF vs. MC^**a**^**	**AF vs. UC^**b**^**	**MC vs. UC^**c**^**
				**AF**	**MC**	**UC**	**OR (95% CI)**	**OR (95% CI)**	**OR (95% CI)**
*IL12A*-504 G/T	Codominant	GG		1 (2.6)	1 (1.1)	0 (0.0)	1		
		GT		13 (33.4)	21 (22.6)	6 (35.3)	0.62 (0.04–10.78)	—	—
		TT		25 (64.0)	71 (76.3)	11 (64.7)	0.35 (0.02–5.84)	—	—
	Dominant	GG		1 (2.6)	1 (1.1)	0 (0.0)	1		
		GT + TT		38 (97.4)	92 (98.9)	17 (100.0)	0.41 (0.03–6.78)	—	—
	Recessive	GG + GT		14 (36.0)	22 (23.7)	6 (35.3)	1	1	1
		TT		25 (64.0)	71 (76.3)	11 (64.7)	0.55 (0.25–1.24)	0.97 (0.3–3.2)	1.76 (0.58–1.76)
			G	15 (19.2)	23 (12.4)	6 (17.6)	1	1	1
			T	63 (80.8)	163 (87.6)	28 (82.4)	0.59 (0.29–1.21)	0.90 (0.32–2.56)	1.52 (0.47–4.06)
*IL18*-607 C/A	Codominant	CC		10 (25.7)	26 (28.0)	8 (47.1)	1		
		CA		16 (41.0)	43 (46.2)	9 (52.9)	0.97 (0.38–2.45)	—	—
		AA		13 (33.3)	24 (25.8)	0 (0.0)	1.41 (0.52–3.80)	—	—
	Dominant	CC		10 (25.7)	26 (28.0)	8 (47.1)	1	1	1
		CA + AA		29 (74.3)	67 (72.0)	9 (52.9)	1.13 (0.48–2.63)	2.58 (0.78–8.5)	2.29 (0.80–6.58)
	Recessive	CC + CA		27 (66.7)	69 (74.2)	17 (100.0)	1		
		AA		13 (33.3)	24 (25.8)	0 (0.0)	1.38 (0.63–3.11)	—	—
			C	36 (46.2)	95 (51.1)	25 (73.5)	1	1	1
			A	42 (53.8)	91 (48.9)	9 (26.5)	1.19 (0.69–2.07)	3.10 (1.24–7.77)	2.61 (1.14–5.96)
*IFNGR1*-611 A/G	Codominant	AA		16 (41.0)	39 (41.9)	7 (41.2)	1	1	1
		AG		19 (48.7)	37 (39.8)	10 (58.8)	2.82 (0.84–9.52)	0.84 (0.2–3.52)	0.64 (0.19–2.18)
		GG		4 (10.3)	17 (18.3)	0 (0.0)	0.39 (0.03-4.22)	—	—
	Dominant	AA		16 (41.0)	39 (41.9)	7 (41.2)	1	1	1
		AG + GG		23 (59.0)	54 (85.1)	10 (58.8)	1.95 (0.61–6.22)	0.97 (0.24–3.92)	0.98 (0.29–3.25)
	Recessive	AA + AG		35 (89.7)	76 (81.7)	17 (100.0)	1		
		GG		4 (10.3)	17 (18.3)	0 (0.0)	0.23 (0.02–2.20)	—	—
			A	51 (65.4)	115 (61.8)	24 (70.6)	1	1	1
			G	27 (34.6)	71 (38.2)	10 (29.4)	0.86 (0.49–1.49)	1.27 (0.53–3.04)	1.48 (0.67–3.38)

a *Reference for AF vs. MC = MC*.

b *Reference for AF vs. UC = UC*.

c *Reference for MC vs. UC = UC*.

Regarding the allelic distribution and frequencies of *IL18*-607 C/A SNP, unconditional logistic regression with adjustments for ethnicity and sex shows significant differences between AF and UC [*p* = 0.015; OR = 3.1 (95% CI: 1.24–7.77)] with a higher frequency of the *A*-allele in AF (53.8%) than in UC (26.5%) (Tables 4, 5). The same is observed in the comparison between MC and UC [*p* = 0.023; OR = 2.61 (95% CI: 1.14–5.96)]. Concerning alleles of *IL12A*-504 G/T and *IFNGR1*-611 A/G SNPs, no statistical differences were found. Furthermore, Table 5 shows the statistical data on sex and ethnicity as covariates of *IL18*-607 C/A SNP in the distribution of genotypes and alleles. There was a significant difference of sex distribution between AF and MC with a higher proportion of women in AF (*p* = 0.000); this is the opposite of the comparison between MC and UC, in which the proportion of female patients is significantly lower in the MC group (*p* < 0.015). This same effect occurred with the codominant, dominant, recessive, and allelic analyses, confirming and detailing the data of Table 2. There were no differences regarding sex distribution between AF and UC, and ethnicity had no statistically significant effect as a covariate in this analysis.

**Table 5:** Association studies between the groups of patients with Acute (AF), Multifocal Chronic (MC), and Unifocal Chronic (UC) forms of PCM including sex, ethnicity, and the *IL18*-607 C/A SNP as covariates and results of *p*-values, Odds Ratio (OR), and 95% Confidence Interval (95% CI).

**Covariates of *IL18*-607 C/A SNP**	**Comparisons**								
	**AF vs. MC**^****c****^			**AF vs. UC**^****d****^			**MC vs. UC**^****e****^		
	***p*-value**	**OR**	**95% CI**	***p*-value**	**OR**	**95% CI**	***p*-value**	**OR**	**95% CI**
Sex^a^	0.000	12.63	3.64–43.81	0.484	1.74	0.37–8.18	0.020	0.16	0.35–0.76
Ethnicity^b^	0.433	1.54	0.52–4.55	0.350	2.12	0.44–10.31	0.517	1.56	0.40–6.00
CC vs. CA vs. AA	0.546	1.25	0.60–2.62	0.070	3.06	0.91–10.27	0.164	1.89	0.77–4.65
Sex^a^	0.000	12.58	3.64–43.50	0.393	1.89	0.44–8.16	0.015	0.151	0.03–0.70
Ethnicity^b^	0.420	1.56	0.53–4.64	0.399	1.85	0.59–9.16	0.600	1.42	0.38–5.36
CC vs. CA + AA	0.755	1.13	0.48–2.63	0.769	2.58	0.78–8.5	0.724	2.29	0.80–6.58
Sex^a^	0.000	12.39	3.58–42.98	0.609	1.52	0.31–7.50	0.018	0.15	0.03–0.72
Ethnicity^b^	0.402	1.59	0.53–4.75	0.699	1.63	0.47–8.00	0.364	1.88	0.48–7.36
CC + CA vs. AA	0.569	1.38	0.63–3.11	Omitted			Omitted		
Sex^a^	0.000	12.6	3.64–43.80	0.484	1.74	0.37–8.31	0.020	0.16	0.03–0.76
Ethnicity^b^	0.430	1.54	0.52–0.30	0.350	2.12	0.44–10.31	0.517	1.56	0.40–6.00
C vs. A	0.528	1.19	0.69–2.07	0.015	3.10	1.24–7.77	0.023	2.61	1.14–5.96

a *Reference for sex = male*.

b *Reference for ethnicity = white*.

c *Reference for AF vs. MC = MC*.

d *Reference for AF vs. UC = UC*.

e *Reference for MC vs. UC = UC*.

## Discussion

Genetic studies are relevant to understanding the mechanisms involved in the pathogenesis of diseases (50, 51). Some genetic polymorphisms are shown to directly interfere in cytokine expression, therefore directing the immune response of the host and possibly influencing the outcome of the disease.

In the present study, we investigated the possible association between the *IL12A*-504 G/T, *IL18*-607 C/A, and *IFNGR1*-611 A/G SNPs in Brazilian patients with PCM and disease susceptibility. The distribution of genotypes and alleles of the *IL12A*-504 G/T SNP was similar in all evaluated comparisons on our study. Although this SNP was related to immune responses against rubella vaccination and HBV and protection in tuberculosis, we did not observe a clear association with PCM (52–54).

As for *IFNGR1*-611 A/G SNP, genotypic and allelic distributions were similar in all evaluated comparisons, confirmed by unconditional logistic regression analysis (adjusted for sex and race). The absence of association with risk or protection reported here is similar to the data reported in tuberculosis and liver fibrosis progression due to recurrent hepatitis C (55–58). An association between tuberculosis and the *IFNGR1*-611 A/G and -56 T/C haplotype was observed by Bullak-Kardum et al. (41). In fact, the promoter activity is supposed to be stronger in -611 A/G than -56 T/C, and the variant *A* is estimated to decrease the binding of GATA-1 and TFIID factors to this site (40, 58). Because previous associations with risk or protection have been described in other SNPs, further studies should include the *IFNGR1*-611 A/G SNP.

In our analysis of the *IL18*-607 C/A SNP, we found no differences on allelic and genotypic distributions between patients and controls or between AF and CF groups of patients. On the other hand, the adjustment for sex and ethnicity in the unconditional regression logistic analysis confirmed the presence of the *A*-allele as a risk factor for the AF and MC groups when compared to the UC group.

The association of the *A*-allele/*AA*-genotype and higher risk is shown in various infectious diseases, such as chronic hepatitis B in Thailand and in India, gingivitis in the Czech Republic, pulmonary tuberculosis in China, Chagas disease in Colombia (mainly driven by rs360719), and infection by the hepatitis C virus in Egypt (37, 43, 52–62). Similarly to our results, the mutant allele/genotype of *IL18*-607 C/A is also associated with more severe outcomes in other infectious diseases: higher virus shedding of the severe acute respiratory syndrome-associated (SARS) coronavirus in Taiwan, lipodystrophy syndrome on HIV-positive Brazilian individuals, immune restoration disease on HIV–tuberculosis coinfected Indian patients, bacterial infections after liver transplantation in China, and hepatitis C–related hepatocellular carcinoma in Egypt (63–67).

The effect of the *A*-allele/*AA*-genotype on PCM and other infectious diseases could be explained by changes in IL-18 levels introduced by this mutant allele in the -607 position. In effect, the IL-18 human gene is composed of six exons and five introns with three very well-known SNPs in the promoter region: -656 G/T (rs1946519), -607 C/A (rs1946518), and -137 G/C (rs187238). Two of these positions, -607 and -137, are thought to be nuclear factor binding sites for cAMP responsive element binding protein and H4TF-1 nuclear factor, respectively, and mutation on both sites can affect the IL-18 levels (50).

The -607 *C*-allele/*CC*-genotype carriage has been associated with higher levels of IL-18 in the serum and/or of mRNA expression (50, 66, 68–70). In our study, we found higher *C*-allele carriage on patients with the UC form of PCM (73.5%), whereas the AF and MC groups (with 46.2 and 51.1% of *C*-allele carriage, respectively) had already been reported to have higher serum IL-18 levels than those from the former group (19, 20).

We hypothesize that the higher *C*-allele carriage on patients with the UC form of PCM may induce higher IL-18 levels at the early stages of infection, determining increased levels of IFN-γ and a more efficient cellular response that controls fungal dissemination and the consequential tissular inflammation. In parallel, previous work in our lab shows that phytohemagglutinin-stimulated cells from *IL18*-607 *C*-allele-unifocal patients apparently produce more IFN-γ than *C*-allele carriers from the acute or the chronic multifocal groups, although without statistically significant difference (unpublished data). However, because both infection and disease in humans are recognized in a later and undefined period after fungal entrance, those initial events can only be evaluated in experimental PCM and have been elegantly shown in deficient mice models. The intravenous infection of IL-18-deficient mice (*IL-18*^−/−^) with *P. brasiliensis* yeast cells resulted in a higher fungal burden in the lungs compared with wild-type (WT) animals and absence of granuloma formation (71). Furthermore, *P. brasiliensis* intratracheally infected mice deficient for NLRP3 inflammasome components (*Nlrp3*^−/−^, *Casp1/11*^−/−^, *Asc*^−/−^) as well as deficient for the ATP receptor (*P2x7r*^−/−^) also had a higher fungal burden in their lungs and liver; predominance of CD4^+^IL-4^+^, CD4^+^TGF-β^+^, and T-reg cells; a lower number of pulmonary PMN cells; and less IL-18 and IL-1β compared with their WT controls (7).

In this context, it is possible that patients with the less severe UC form of PCM present a more balanced IL-18/IL-12/IFN-γ axis, resulting in more localized and milder clinical manifestations compared with the other groups, in which the higher IL-18 levels are accompanied by other immunomodulating cytokines. The more severe form of PCM, the acute form, has been characterized by IL-4 with IL-18 inducing a Th2 immune response (high levels of IL-4, IL-5, and IL-13), by IL-4 and TGF-β inducing a Th9 pattern (high levels of IL-9), and both Th2 and Th9 inhibiting the Th1 response. Patients with chronic PCM were previously shown to have a mixed immune response of Th1 (IL-12 and IL-18 leading to IFN-γ production), Th17 (induced by IL-18, IL-1 and IL-23), and Th22 (induced by IL-18, IL-1, IL-23, and IL-21), which result in heterogeneous clinical symptoms (12, 13, 20).

In our study, the UC group included only patients with mild and restricted disease to the skin, mucosae, or lymph nodes. The exclusion of patients with lung involvement was based on the possible misclassification of MC patients who are more prone to develop pulmonary lesions. Although these criteria resulted in fewer patients in the UC group, it also revealed specific immunogenetic characteristics, such as the association of a more favorable outcome with the *IL18*-607 *C*-allele in the present study and the *IL12RB1* 641 *AG*-genotype and the HLA-DRB1^*^11 allele carriage, previously reported by our group (26, 31).

Additionally, the association of the *IL18*-607 *A*-allele with the more severe forms of PCM described herein collaborates with previous reports on different genes emphasizing the influence of genetic background on the outcome of this mycosis. The *GG*-genotype of *IL10*-1082 G/A SNP, the *RP2/RP2*-genotype of the intron-3 microsatellite polymorphism of the *IL4* gene, the *AA*-genotype of *IL12RB1* 641 A/G SNP, the *GG*-genotype of the *DCSIGN* rs4804803 A/G SNP, and the *CC*-genotype of the *VDR* rs7975232 A/C SNP have all been previously associated with susceptibility or the more severe outcome in PCM (26, 29–31, 34, 35, 49). In parallel, HLA class I antigens and the GLO-1 phenotype of the glyoxalase enzyme have been associated with the progression of disease (pulmonary form) or infection (23–25, 27, 28).

We reinforce that our present data and all the genetic associations previously described may be considered in the same context of association of autosomal gene dominance with resistance in murine PCM, which is a model that reproduces several characteristics of the human disease (22).

The comparison between patients and controls in our study shows a greater number of male than female subjects, particularly in the MC group. As previously described, there has been a male predominance among cases of chronic PCM (72–74). This mycosis manifests more frequently in male farmers who are constantly working in direct contact with the soil where *Paracoccidioides* spp. probably occurs (1). In addition, infected women are less likely to manifest the chronic form because of the putative role of estrogen (17-β-estradiol) as a protective factor that impairs conidia transformation into yeast form during murine infection (75–77). Although this effect has been less commonly registered in human PCM, epidemiological data reported in Brazil show that the disease is rare in adult females (4.3%) and usually occurring in the menopausal period (91.3%) (78). Contrarily, it has been demonstrated that 17-β-estradiol can exert an anti-inflammatory role by decreasing TNF-α and IL-6 while increasing IL-4 levels, which further results in a Th2 response (79). In oral PCM lesions, a positive correlation between the amount of estradiol receptors and the fungal burden was observed only in female patients (80). These recent findings may explain the more even distribution of the acute form of PCM among sexes, and the higher frequency of females in UC and lower in MC could be evidence of protection for women in developing the more severe chronic form of the disease.

Possible limitations in our study are the inclusion of only one health center, mixed ethnic groups in the Brazilian population, and lack of detection of IL-18 levels and functional analyses. Although reflecting the distribution of PCM in endemic areas, our cohort with a low number of patients with the UC form is also a limitation for further inquiry.

In summary, our study did not show an association between PCM and the evaluated *IL12A* and *IFNGR1* SNPs or between the acute and chronic forms or between multifocal and unifocal chronic forms. For the *IL18*-607 C/A SNP, no association was shown with infection or among acute and chronic forms. However, we show an association between the *IL18*-607 *A*-allele and the more severe clinical forms of PCM, acute and multifocal chronic forms, when compared to the UC form, the less severe form of this disease, associated with the *IL18*-607 *C*-allele. To the best of our knowledge, this is the first study that evaluates the association between *IL12A, IFNGR1*, and *IL18* SNPs and PCM.

The present data suggest a novel role of *IL18*-607 C/A SNP as a contributor to a more favorable outcome of this disease, potentially leading to a more balanced and more efficient cellular response for the control of fungal dissemination at the early stages of infection in UC. Furthermore, our work highlights the need for new studies with other SNPs on the *IL18* gene and on other components of the immune response for a better understanding of the pathophysiology and the clinical expression of PCM.

## Data Availability Statement

The datasets generated for this study are available on request to the corresponding author.

## Ethics Statement

The studies involving human participants were reviewed and approved by Research and Ethics Committee of Hospital das Clínicas, Faculdade de Medicina, University of São Paulo - HCFMUSP, São Paulo, SP, Brazil (CAPPesq 10273; Plataforma Brasil 123334/2013). The patients/participants provided their written informed consent to participate in this study.

## Author Contributions

PS and MS-Y conceived and designed the experiments. MM and AK contributed with samples and patient selection. PS, FB, FC, CD, and AS performed the experiments. PS, AG, and OL analyzed the data. PS, FB, AG, FC, and MS-Y wrote the paper and/or performed bibliographic revision. PS, MS-Y, MM, AK, FB, FC, CD, AS, AG, and OL approved the paper. All authors contributed to the article and approved the submitted version.

## Conflict of Interest

The authors declare that the research was conducted in the absence of any commercial or financial relationships that could be construed as a potential conflict of interest.
