# Supplementary material for: Polymorphism in the Promoter Region of the IL18 Gene and the Association With Severity on Paracoccidioidomycosis
Source: Front Immunol. 2020 Oct 1;11:542210. doi: 10.3389/fimmu.2020.542210 (PMC7559583; doi:10.3389/fimmu.2020.542210)
Supplement: Supplementary file 1 [file Data_Sheet_1.PDF]

Table S1 – Polymorphisms on *IL18* (-607 C/A, rs1946518 and -137 G/C, rs187238) associated with infectious diseases (Medline indexed journals; SNP and rs as informed by the respective article)

IL18 SNPs	Morbidity	Country / Population	Association with	Main Results	Ref.
-607 C/A and −137 G/C	Post-injury Sepsis	USA	Protection	Haplotype -607 CA/-137 GC genotype was protective against sepsis development, suggesting a role in the predisposition for the development of post-injury sepsis.	1
-607 C/A and −137 G/C	Chronic HBV Infection	China	Protection	Haplotypes -607C/-137C and -607A/-137C in Chronic HBV groups were lower than in controls. Frequency of -607 AA genotype in high HBV-DNA copies groups was lower than that in low HBV-DNA copies groups and suggested the role of 607 AA in virus replication inhibition.	2
-607 G/T (rs1946518)	Severe Acute Respiratory Syndrome (SARS) - Coronavirus	Taiwan	Susceptibility	The homozygous individuals TT of 607 had increased risk of virus shedding, compared with the heterozygous individuals CA when genotypes of the other 3 alleles were controlled.	3
−607 C/A and −137 G/C	HIV-1	Brazil	Susceptibility	The -607C allele and -607C/-137G haplotype are more frequent in HIV+ than on controls [*p*= 0.02; OR= 1.59 (95% CI: 1.07-2.37)].	4
−607 A/C and −137 G/C	Chronic HBV Infection	Thailand	Susceptibility	There was a significant involvement of genotype AA at position -607 in patients when compared with healthy individuals [*p*= 0.009; OR= 2.62 (95% CI: 1.36–5.09)].	5
-607 C/A and −137 G/C	HCV Clearance	European-American and African-American	Protection	Among African-American patients, the 607A allele occurred more frequently in the clearance group than in the persistence group (*p* <0.001), and in a dominant genetic model, the genotypes carrying 1 or 2 copies of 607A [*p*= 0.001; OR= 2.92 (95% CI 1.59–5.36)]. Among injection drug users, 607A was significantly associated with HCV clearance [OR= 3.68 (95% CI: 1.85–7.34)]. A haplotype carrying 607A and 137C [OR= 4.53 (95% CI: 1.77–11.6)] was also strongly associated with viral clearance.	6
−607 C/A and −137 G/C	Chronic HCV Infection	Tunisia	Susceptibility	The carriage of at least one C allele at position -607 (CC + CA) was associated with a higher risk of cirrhosis and Hepatocellular Carcinoma (HCC) (*p*= 0.032). Compared with controls, HCV-infected patients had significantly higher levels of IL-18 (*p*= 0.0001) that correlate with disease severity (*p*= 0.01 [chronic hepatitis], *p*= 0.001 [cirrhosis], *p*= 0.0006 [HCC]). No real significant differences in IL-18 levels were found between patients with 607 SNP AA genotype compared to CC+AC genotypes, *p*= 0.072 ("AA lower producer").	7
−607 C/A and −137 G/C	*H. pylori*-infected gastric mucosa	Japan	Susceptibility	*H. pylori*–infected patients displaying -607CC and -137GG had higher IL-18 levels than did those with other genotypes and were more likely to experience treatment failure.	8
−607 C/A and −137 G/C	HCV	European Population	Protection	In viral genotype 1, the -607A allele was positively associated with treatment response.	9

−607 C/A and −137 G/C	Liver disease severity in HCV Infection	India	Protection	The -607 A/A allele was more common in group A patients with mild liver disease than in patients with severe liver disease on the basis of HAI [38.6% vs. 21%; *p*= 0.05; OR= 0.424 (95% CI: 0.233–0.773)] and stage of fibrosis [38.7% vs. 16.7%; *p*= 0.008; OR= 0.282 (95% CI: 0.134–0.596)].	10
-607 A/C (rs1946518) and -137 C/G (rs187238)	HBV-related liver disease	Japan	Protection	In these HBV-infected patients, the frequencies of AA genotype at position -607 and C allele at position -137 were significantly higher in inactive HBV carriers compared with those in patients with chronic progressive liver disease.	11
-607 A/C	Gingivitis	Czech Republic	Susceptibility	Patients with -607 CC genotype had a 5.61-fold increased risk of gingivitis as compared with a subject without this combination.	12
−607 C/A and −137 G/C	HIV and Lipodystrophy (LS)	Brazil	Susceptibility	The −607A allele, −607AA genotype, and −137G/–607A and −137C/–607A haplotypes were over-represented in HIV patients presenting LS.	13
			Protection	The −137G/−607C haplotype was associated with protection against LS.	
-607 C/A (rs1946518) and -137 G/C (rs187238)	Severe Malarial Anemia (SMA)	Kenia	Susceptibility	Carriers of the -137G/-607C (GC) haplotype had increased susceptibility to SMA [*p*= 0.039; OR= 2.05 (95% CI: 1.04-4.05)] and elevated IL-18 transcripts (*p* = 0.026) with reduced hemoglobin concentrations (*p* = 0.010). Longitudinal investigation of clinical outcomes over a three year follow-up period revealed that carriers of the rare CC haplotype (~1% frequency) had 5.76 times more mortality than non-carriers (*p* = 0.001). Carriage of the rare CC haplotype significantly increases the risk of childhood mortality.	14
			Protection	The carriage of the -607 AA genotype was associated with protection against SMA [*p*= 0.031; OR= 0.44 (95% CI: 0.21-0.90)] in children with acute infection.	
**rs1946518 C/A**, rs5744247, and rs549908	TB	China (Han)	Susceptibility	Allele A of rs1946518 confers a 1.47-fold increased risk of developing TB [*p*= 0.0001; OR= 1.47 (95% CI: 1.21–1.78)]. Estimation of the frequencies of haplotypes revealed a potential risk haplotype AGA [*p*= 0.01; OR= 1.41 (95% CI: 1.15–1.72)].	15
			Protection	The haplotype CCA was protective for TB [*p*= 0.01; OR= 0.70 (95% CI: 0.57–0.85)].	
-607 C/A and −137 G/C	HCV Infection	Tunisia	Protection	Multivariate analysis: -137GC and -607CA genotypes, and haplotype GC-CA were associated with spontaneous clearance of HCV.	16
−607 C/A	HIV-1	North India	Protection	The −607A allele may play a protective role against the progression of HIV-1 infection in this population.	17
rs1946518 A/C and rs574424 C/G	Chronic HBV Infection	China	Susceptibility	In the genotypes of rs1946518, the AA type was present at a higher frequency in the patients compared to those in the controls. OR of the AA genotype for the comparison with that of the AC and the CC genotype was 1.537 (95% CI: 1.116-2.218; *p*= 0.009). OR of the allele A for the comparison with that of the allele C was 1.279 (95% CI: 1.045-1.567). The data suggest that genotype AA and the allele A are closely associated with the resistance to chronic hepatitis B and may be the dangerous gene (*p* <0.05).	18
			Protection	In phenotypes, the allele C at rs1946518 was of a significantly lower frequency in the patients with chronic hepatitis B than that in the controls (*p*= 0.017).	

−607 A/C and −137 C/G	Human T-cell Lymphotropic Virus type 1 (HTLV-1)	Brazil	Susceptibility<br>Protection	The −607AC genotype was significantly higher in HAC and HTLV-1-infected individuals group compared to the healthy control group. The −137G/−607A haplotype was higher in infected group than healthy control group.<br>The −607CC genotype was significantly lower in HTLV-1 asymptomatic carriers and HTLV-1-infected individuals than healthy control group. The −137C/−607C haplotype was increased in the healthy control group compared to the others.	19
-607 T/G and −137 G/C	HIV, TB and Immune Restoration Disease (IRD)	Cambodia and India	Susceptibility	Susceptibility to Tuberculosis-IRD was associated with carriage of -607 G allele [*p*= 0.02; OR= 3.8 (95% CI: 1.2-12)] in Indian patients.	20
-607 C/A and −137 G/C	*H. pylori*-infected duodenal ulcer	Iran	Susceptibility<br>Protection	Increase in the CC genotype and decrease in AA genotype/A Allele frequencies in duodenal ulcer patients. The haplotype 137G/607C was more frequent in patients than in asymptomatic carriers and *H. pylori*-negative subjects. In the duodenal ulcer patients the 607 CC genotype group showed an increased IL-18 serum level compared with those of the AA genotype.<br>The genotype combination 137GG/607AA was lower in duodenal ulcer patients compared with asymptomatic carriers and *H. pylori* negative subjects.	21
-607 C/A (rs1946518)	Therapy for HCV Infection	Pakistan	Protection	The distribution of the -607AA genotype was significantly high in healthy subjects than HCV patients (*p*= 0.031), signifying its potential involvement in the natural clearance of HCV infection. The occurrence of the -607AA genotype was also significantly higher in the sustained virological group than in the nonresponder group (*p*= 0.046), highlighting its protective involvement in the treatment outcome of chronic HCV infection.	22
−607 C/A and −137 G/C	HBV-related Hepatocellular Carcinoma	China	Protection	The −607A/−137C haplotype was associated with a significant decreased risk of Hepatocellular Carcinoma [*p*= 0.007; OR= 0.495 (95% CI= 0.294–0.834)].	23
−607 A/C and −137 G/C	HBV-related liver disease	India	Susceptibility<br>Protection	The genotype CC at position -607 in cases (18.0%) when compared to healthy controls (6.7%) [OR= 3.03 (95% CI: 1.734–5.303)] also associated with persistent HBV infections (24.1%) compared to spontaneous clearance (9.2%) [OR= 0.31 (95% CI: 0.151–0.67)].<br>The results demonstrated the significant involvement of genotype AA at position -607 in healthy controls (38.6%) when compared to cases (26.0%) [OR= 0.54 (95% CI= 0.385–0.797)] and also associated with spontaneous clearance (37.6%) compared to persistent HBV in infections (17.9%) [OR= 2.76 (95% CI: 1.582–4.832)].	24
-607 C/A (rs1946518) and −137 G/C (rs187238)	Persistent HCV Infection	Brazil	Susceptibility	Comparison of polymorphism allele frequencies between the patient and control groups showed a higher frequency of C allele at position -607 among patients (*p*= 0.02). When the association between the polymorphisms and viral infection was analyzed, patients carrying genotype CA at position -607 were found to be at higher risk of persistent HCV infection (*p*= 0.03).	25
-607 C/A and −137 G/C	TB	China	Protection	The frequency of -137C/-607C haplotype in TB patients group was decreased when compared with that in the control group.	26

rs5744258, rs360722, rs2043055, **rs187238, rs1946518** and rs360719	Chagas Disease	Colombia	Susceptibility	rs1946518-C was reduced in the seronegative sample set in comparison with the seropositive one (*p*= 0.0162, PFDR= 0.0243; OR= 1.22; 95% CI: 1.04–1.44), indicating that it is associated with a higher infection risk. Significant associations with *Trypanosoma cruzi* infection were observed when comparing seronegative and seropositive individuals for rs187238, rs360719, rs2043055 and rs1946518, but mainly driven by rs360719.	27
-607 C/A (rs1946518) and −137 G/C (rs187238)	Human Papilloma Virus (HPV) Infection	Brazil	Protection	The -607A allele conferred protection against HPV infection (*p*= 0.0043). The *IL18* SNPs were not associated with development of cervical lesions (*p* >0.05).	28
-607 C/A (rs1946518) and −137G/C (rs187238)	Pulmonary TB with co-morbid Diabetes Mellitus	India	Susceptibility	At -607 C/A variant, a positive association was shown with AC genotype in TB + Diabetes, their "families" and Diabetes.	29
rs7106524, rs5744247, **rs1946518**, rs549908 and **rs187238**	Infection after liver transplantation	China	Susceptibility	IL-18 mRNA mean expression levels and protein levels were significantly lower in liver transplant patients with bacterial infections. For the donor SNP rs1946518, more recipients carried the A allele in the bacterial-infected group than the uninfected group (61.4% vs 39.7%; *p* ≤0.002). The mean IL-18 mRNA expression and protein levels were significantly lower in the transplanted livers of recipients carrying the rs1946518 AA genotype compared with those from recipients with CC genotype (3.64, 3.33 vs. 2.75, *p* ≤0.048). The area under ROC curve indicated that the rs1946518 SNP genotype in the donor liver predicted an increased risk of bacterial infection after liver transplantation (AUROC >0.82).	30
-607 C/A and −137 G/C	Periodontitis	Japan	Protection	The -607CC genotype had a significantly reduced risk of periodontal disease.	31
-607 C/A (rs1946518) and −137 G/C (rs187238)	HCV-related Hepatocellular Carcinoma and Metastasis	Egypt	Susceptibility	The -607A allele and AA genotype were significantly related to a higher risk of developing Hepatocellular Carcinoma (HCC) when comparing patients with HCC and controls, and were significantly related to a higher risk of metastasis when comparing metastatic and nonmetastatic groups. A significant association was found between multinodular HCC and -607AA genotype.	32
rs1946518 G/T and rs187238 C/G	HCV infection and outcome of Interferon treatment	Egypt	Susceptibility<br><br>Protection	The rs1946518-T allele was associated with increased risk of HCV positivity.<br><br>Genotype GG and allele G (rs1946518): more frequent in the healthy control.	33

Abbreviations: OR: Odds Ratio; 95% CI: 95% Confidence Interval; HBV: Hepatitis B Virus; HIV: Human Immunodeficiency Virus; HCV: Hepatitis C Virus; *H. pylori*: *Helicobacter pylori*; TB: Tuberculosis.

References:

1. Stassen NA, Breit CM, Norfleet LA, Polk HC Jr. IL-18 promoter polymorphisms correlate with the development of post-injury sepsis. Surgery (2003) 134:351–6. doi:10.1067/msy.2003.248
2. Zhang PA, Wu JM, Li Y, Yang XS. Association of polymorphisms of interleukin-18 gene promoter region with chronic hepatitis B in Chinese Han population. World J Gastroenterol (2005) 11:1594–8. doi:10.3748/wjg.v11.i11.1594
3. Chen W-J, Yang J-Y, Lin J-H, Fann CSJ, Osyetrov V, King C-C, et al. Nasopharyngeal shedding of severe acute respiratory syndrome-associated coronavirus is associated with genetic polymorphisms. Clin Infect Dis (2006) 42:1561–9. doi:10.1086/503843
4. Segat L, Bevilacqua D, Boniotto M, Arraes LC, de Souza PR, de Lima Filho JL, et al. IL-18 gene promoter polymorphism is involved in HIV-1 infection in a Brazilian pediatric population. Immunogenetics (2006) 58:471–3. doi:10.1007/s00251-006-0104-7
5. Hirankarn N, Manonom C, Tangkijvanich P, Poovorawan Y. Association of interleukin-18 gene polymorphism (-607A/A genotype) with susceptibility to chronic hepatitis B virus infection. Tissue Antigens (2007) 70:160–3. doi:10.1111/j.1399-0039.2007.00865.x
6. An P, Thio CL, Kirk GD, Donfield S, Goedert JJ, Winkler CA. Regulatory polymorphisms in the interleukin-18 promoter are associated with hepatitis C virus clearance. J Infect Dis (2008) 198:1159–65. doi:10.1086/592047
7. Bouzgarrou N, Hassen E, Schvoerer E, Stoll-Keller F, Bahri O, Gabbouj S, et al. Association of interleukin-18 polymorphisms and plasma level with the outcome of chronic HCV infection. J Med Virol (2008) 80:607–14. doi:10.1002/jmv.21079
8. Sakai K, Kita M, Sawai N, Shiomi S, Sumida Y, Kanemasa K, et al. Levels of interleukin-18 are markedly increased in *Helicobacter pylori*-infected gastric mucosa among patients with specific *IL18* genotypes. J Infect Dis (2008) 197:1752–61. doi:10.1086/588196
9. Haas SL, Weiss C, Bugert P, Gundt J, Witt H, Singer MV, et al. Interleukin 18 promoter variants (-137G>C and -607C>A) in patients with chronic hepatitis C: association with treatment response. J Clin Immunol (2009) 29:620–8. doi:10.1007/s10875-009-9302-z
10. Manohar K, Suneetha PV, Sukriti, Pati NT, Gupta AC, Hissar S ,et al. Association of IL-18 promoter polymorphism with liver disease severity in HCV-infected patients. Hepatol Int (2009) 3:371–7. doi:10.1007/s12072-009-9127-0
11. Migita K, Sawakami-Kobayashi K, Maeda Y, Nakao K, Kondoh S, Sugiura M, et al. Interleukin-18 promoter polymorphisms and the disease progression of Hepatitis B virus-related liver disease. Transl Res (2009) 153:91–6. doi:10.1016/j.trsl.2008.11.008
12. Vokurka J, Klapusová L, Pantuckova P, Kukletova M, Kukla L, Holla LI. The association of MMP-9 and IL-18 gene promoter polymorphisms with gingivitis in adolescents. Arch Oral Biol (2009) 54:172–8. doi:10.1016/j.archoralbio.2008.09.002
13. Castelar L, Silva MM, Castelli EC, Deghaide NHS, Mendes-Junior CT, Machado AA, et al. Interleukin-18 and interferon-gamma polymorphisms in Brazilian human immunodeficiency virus-1-infected patients presenting with lipodystrophy syndrome. Tissue Antigens (2010) 76:126–30. doi:10.1111/j.1399-0039.2010.01471.x
14. Anyona SB, Kempaiah P, Raballah E, Ouma C, Were T, Davenport GC, et al. Functional promoter haplotypes of interleukin-18 condition susceptibility to severe malarial anemia and childhood mortality. Infect Immun (2011) 79:4923–32. doi:10.1128/IAI.05601-11
15. Han M, Yue J, Lian YY, Zhao YL, Wang HX, Liu LR. Relationship between single nucleotide polymorphism of interleukin-18 and susceptibility to pulmonary tuberculosis in the Chinese Han population. Microbiol Immunol (2011) 55:388–93. doi:10.1111/j.1348-0421.2011.00332.x
16. Ksiaa Cheikhrouhou L, Sfar I, Aounallah-Skhiri H, Aouadi H, Jendoubi-Ayed S, Abdallah TB, et al. Cytokine and apoptosis gene polymorphisms influence the outcome of hepatitis C virus infection. Hepatobiliary Pancreat Dis Int (2011) 10:280–8. doi:10.1016/s1499-3872(11)60047-7
17. Sobti R, Sharma V, Abitew A, Berhane N, Mahdi S, Askari M, et al. IL-18 Gene Promoter Region 607C/A Polymorphism in HIV-1 Infected North Indian Population. Balkan J Med Genet (2011) 14:41–8. doi:10.2478/v10034-011-0046-8
18. Li N, Gao YF, Zhang TC, Chen P, Li X, Su F. Relationship between interleukin 18 polymorphisms and susceptibility to chronic hepatitis B virus infection. World J Hepatol (2012) 4:105–9. doi:10.4254/wjh.v4.i3.105

19. Rocha-Júnior MC, Haddad R, Cilião Alves DC, de Deus Wagatsuma VM, Mendes-Junior CT, Deghaide NHS, et al. Interleukin-18 and interferon-gamma polymorphisms are implicated on proviral load and susceptibility to human T-lymphotropic virus type 1 infection. Tissue Antigens (2012) 80:143–50. doi:10.1111/j.1399-0039.2012.01887.x

20. Affandi JS, Kumar M, Agarwal U, Singh S, Price P. The search for a genetic factor associating with immune restoration disease in HIV patients co-infected with *Mycobacterium tuberculosis*. Dis Markers (2013) 34:445–9. doi:10.3233/DMA-130991

21. Rezaeifar A, Eskandari-Nasab E, Moghadampour M, et al. The association of interleukin-18 promoter polymorphisms and serum levels with duodenal ulcer, and their correlations with bacterial CagA and VacA virulence factors. Scand J Infect Dis (2013) 45:584–92. doi:10.3109/00365548.2013.794301

22. Imran M, Manzoor S, Parvaiz F. Predictive potential of IL-18 -607 and osteopontin -442 polymorphism in interferon-based therapy of HCV infection in the Pakistani population. Viral Immunol (2014) 27:404–11. doi:10.1089/vim.2014.0044

23. Bao J, Lu Y, Deng Y, Rong C, Liu Y, Huang X, et al. Association between IL-18 polymorphisms, serum levels, and HBV-related hepatocellular carcinoma in a Chinese population: a retrospective case-control study. Cancer Cell Int (2015) 15:72. doi:10.1186/s12935-015-0223-z

24. Karra VK, Gumma PK, Chowdhury SJ, Ruttala R, Polipalli SK, Chakravarti A, et al. IL-18 polymorphisms in hepatitis B virus related liver disease. Cytokine (2015) 73:277–82. doi:10.1016/j.cyto.2015.02.015

25. Santos KN, Almeida MK, Fecury AA, Costa CA, Martins LC. Analysis of polymorphisms in the interleukin 18 gene promotor (-137 G/C and -607 C/A) in patients infected with hepatitis c virus from the Brazilian amazon. Arq Gastroenterol (2015) 52:222–7. doi:10.1590/S0004-28032015000300013

26. Zhou C, Ouyang N, Li QH, Luo SX, He Q, Lei H, et al. The -137G/C single nucleotide polymorphism in IL-18 gene promoter contributes to tuberculosis susceptibility in Chinese Han population. Infect Genet Evol (2015) 36:376–80. doi:10.1016/j.meegid.2015.10.014

27. Leon Rodriguez DA, Carmona FD, Echeverría LE, González CI, Martin J. IL18 Gene Variants Influence the Susceptibility to Chagas Disease. PLoS Negl Trop Dis (2016) 10:e0004583. doi:10.1371/journal.pntd.0004583

28. Tavares MC, de Lima Júnior SF, Coelho AV, Marques TRNM, de Araújo DHT, Heráclio SA, et al. Tumor necrosis factor (TNF) alpha and interleukin (IL) 18 genes polymorphisms are correlated with susceptibility to HPV infection in patients with and without cervical intraepithelial lesion. Ann Hum Biol (2016) 43:261–8. doi:10.3109/03014460.2014.1001436

29. Ponnana M, Sivangala R, Joshi L, Valluri V, Gaddam S. IL-6 and IL-18 cytokine gene variants of pulmonary tuberculosis patients with co-morbid diabetes mellitus and their household contacts in Hyderabad. Gene (2017) 627:298–306. doi:10.1016/j.gene.2017.06.046

30. Shi BJ, Yu XY, Li H, Xing T-H, Fan J-W, Wang P-W, et al. Association between donor and recipient Interleukin-18 gene polymorphisms and the risk of infection after liver transplantation. Clin Invest Med (2017) 40:E176–87. doi:10.25011/cim.v40i5.28623

31. Tanaka K, Miyake Y, Hanioka T, Furukawa S, Miyatake N, Arakawa M. The *IL18* Promoter Polymorphism, rs1946518, Is Associated with the Risk of Periodontitis in Japanese Women: The Kyushu Okinawa Maternal and Child Health Study. Tohoku J Exp Med (2017) 243:159–64. doi:10.1620/tjem.243.159

32. Bakr NM, Awad A, A Moustafa E. Association of genetic variants in the interleukin-18 gene promoter with risk of hepatocellular carcinoma and metastasis in patients with hepatitis C virus infection. IUBMB Life (2018) 70:165–74. doi:10.1002/iub.1714

33. Estafnous SZK, Ali SA, Seif SM, Soror SHA, Abdelaziz DHA. Inflammasome genes' polymorphisms in Egyptian chronic hepatitis C patients: influence on vulnerability to infection and response to treatment. Mediators Inflamm (2019) 2019:3273645. doi: 10.1155/2019/3273645

Table S2 – Polymorphism on *IL12A* (- 504 G/T rs2243115) and infectious diseases (Medline indexed journals; SNP and rs as informed by the respective article)

IL12A SNPs	Morbidity	Country / Population	Association with	Main Results	Ref.
**rs2243115 (-564 T>G)**	Rubella vaccine	USA	Susceptibility	Increased carriage of major allele T for rs2243115 (*IL12A*-564 T>G) was associated with a dose-related decrease (p= 0.002) in IL-2 secretion levels.	1
**rs2243115 (T > G)** and rs568408 (G > A)	Pulmonary TB	China	Susceptibility	The genetic variants TG/GG of rs2243115 of *IL12A* were associated with a decreased risk of TB [OR= 0.7 (95% CI: 0.49–0.99)].	2
**rs2243115 (T>G)** and rs568408 (G>A)	HBV infection and risk of HCC	China	No association	No significant associations between *IL12A* rs2243115 T/G and risk of HCC were observed.	3
**rs2243115**	HBV vaccine	China	Susceptibility	The frequency of the combined genotypes *IL12A* rs2243115 TT and *IL12B* rs17860508 CTCTAA/CTCTAA was significantly higher in the low-response group than in the high-response group [p= 0.008, OR= 2.19 (95% CI: 1.23–3.93)].	4
**rs2243115 (T>G)** and rs568408 (G>A)	HBV clearance and HBV-related HCC	China	Susceptibility	The TG haplotype was observed to be associated with a significantly increased risk of HBV-related HCC [OR= 1.42 (95 % CI: 1.10–1.83), p= 0.006].	5
			Protection	The TA haplotype was associated with a decreased risk of HBV-related HCC [OR= 0.61 (95 % CI: 0.45–0.83), p= 0.002].	
**rs2243115** and rs568408	Bacillus Calmette-Guérin Osteitis	Finland	No association	The distributions of *IL12A* rs2243115 SNP did not differ between BCG osteitis patients and Finnish population-based controls.	6

Abbreviations: OR: Odds Ratio; 95% CI: 95% Confidence Interval; TB: Tuberculosis; HBV: Hepatitis B Virus; HCC: Hepatocellular Carcinoma.

References

1. Dhiman N, Haralambieva IH, Kennedy RB, Vierkant RA, O'Byrne MM, Ovsyannikova IG, et al. SNP/haplotype associations in cytokine and cytokine receptor genes and immunity to rubella vaccine. Immunogenetics (2010) 62:197–210. doi:10.1007/s00251-010-0423-6
2. Wang J, Tang S, Shen H. Association of genetic polymorphisms in the *IL12-IFNG* pathway with susceptibility to and prognosis of pulmonary tuberculosis in a Chinese population. Eur J Clin Microbiol Infect Dis (2010) 29:1291-5. doi:10.1007/s10096-010-0985-0
3. Liu L, Xu Y, Liu Z, Chen J, Zhang Y, Zhu J, et al. *IL12* polymorphisms, HBV infection and risk of hepatocellular carcinoma in a high-risk Chinese population. Int J Cancer (2011) 128:1692-6. doi: 10.1002/ijc.25488
4. Pan L, Zhang W, Liang Z, Wu X, Zhu X, Li J, et al. Association between polymorphisms of the cytokine and cytokine receptor genes and immune response to hepatitis B vaccination in a Chinese Han population. J Med Virol (2012) 84:26-33. doi:10.1002/jmv.22251
5. Tan A, Gao Y, Yao Z, Su S, Jiang Y, Xie Y, et al. Genetic variants in *IL12* influence both hepatitis B virus clearance and HBV-related hepatocellular carcinoma development in a Chinese male population. Tumour Biol (2016) 37:6343-8. doi: 10.1007/s13277-015-4520-x
6. Korppi M, Teräsjärvi J, Lauhkonen E, Pöyhönen L, Huhtala H, Nuolivirta K, et al. Interferon-γ and interleukin-12 production in relation to gene polymorphisms in bacillus Calmette-Guérin osteitis. Pediatr Int (2019) 61:982–7. doi:10.1111/ped.13998

Table S3 – Polymorphism on *IFNGR1* (-611 A/G, rs1327474) and infectious diseases (Medline indexed journals; SNP and rs as informed by the respective article)

IFNGR1 SNPs	Morbidity	Country	Association with	Main results	Ref.
+95 T/C, -56 T/C, -270 T/C, **-611 A/G** and -470 Ins/Del	Cerebral/Severe Malaria	Gambia	No association with the -611 A/G SNP	The ethnic group Mandinka, heterozygotes for the *IFNGR1* -56 polymorphism appeared to be protected against Cerebral Malaria (OR= 0.54; *p*= 0:016) and against fatal outcome (OR= 0.22; *p*= 0:006), and those carrying the double deletion at position *IFNGR1* -470 (OR= 0.58; 95% CI: 0.36–0.93; *p*= 0.017) were protected against severe malaria in general.	1
**-611 A/G**, -470 Ins/Del, -270 T/C, -56 T/C, +95 T/C and +189 T/G (exon 7)	Pulmonary TB	Gambia	No association	There was no association between the *IFNGR1* variants studied and TB in this Gambian population sample.	2
**-611 A/G**, -470 Ins/Del, -255 T/C, -56 T/C, -169 A/C and -72 T/C	Pulmonary TB, Non-*M. tuberculosis* Pulmonary Micobacteriosis, Non-*M. tuberculosis* Disseminated Micobacteriosis	USA and Korea	No association with the -611 A/G SNP	There was no association between the *IFNGR1* variants studied and mycobacterial susceptibility in the evaluated population.	3
rs608914, **rs1327474**, rs13247475 and rs3799488	Schistosomal Hepatic Fibrosis	Egypt	No association with rs1327474	There was evidence for linkage of schistosomal hepatic fibrosis to a polymorphism in the *IFNGR1* gene and suggestive evidence for linkage to a locus in the *IL13–IL4* region and *TGFB1*. The rs1327475 marker of *IFNGR1* produced the most significant *p*-value (0.0000013) in model-free single point analysis.	4
**G-611A**, T-56C, 192 FA1(CA)n	Pulmonary TB	Croatia	Protection	The 192 FA1(CA)n heterozygosity [OR= 0.24 (95% CI: 0.08–0.68); *p*= 0.0023] and the -611G/-56T/192 FA1 (CA)22 haplotype [OR= 0.27 (95% CI: 0.06–0.95); *p*= 0.038] were more frequent on controls than on patients.	5
**-611 A/G** and -56 C/T	Liver Fibrosis progression due to recurrent HCV	Italy	No association	There was no association between -611 A/G and -56 C/T SNPs and the evaluated Italian population with Liver Fibrosis progression due to recurrent HCV.	6
**-611 G/A** and -56 T/C	Non-*M. tuberculosis* Pulmonary Micobacteriosis	Korea	No association	The patients with Non-*M. tuberculosis* lung disease showed no significant difference from controls in genotype and allele frequencies of the IFN-gR1 -611 and -56 polymorphisms.	8
**-611 G/A**, -270 T/C, -56 T/C and +95 T/C	TB	Korea	No association	The genotype and allele frequencies of the IFN-gR1 gene polymorphisms did not differ significantly between the patients with pulmonary TB and controls.	7

**rs1327474**, rs2234711 and rs4896243	Pulmonary TB	Uganda	No association with rs1327474	Both rs2234711 and rs4896243 alleles were associated with increased risk for TB and increased TNF-α expression.	9
**rs1327474**	Pulmonary TB	USA and Argentina	Susceptibility	There was an elevated susceptibility on African-American descendants to TB in the presence of both *IFNGR1* rs1327474 AA and the minor allele of four of the *NOS2A* SNPs, with ORs ranging 1.61–2.47.	10
**–611 A/G**, –56 C/T, 40 G/A, 95 C/T, 130 A/G, 20685 A/G, 21227 T/C	Chronic HBV Infection	China	No association with the -611 A/G SNP	The –56T allele was associated with viral persistence (*p*= 0.014), and the –56C allele were associated with viral clearance (*p*= 0.014); the promoter variant with –56C exhibited a higher transcription level than that with –56T in HepG2 cells.	11
rs2234711, **rs1327474**, rs7749390 and rs41401746	Pulmonary TB	China	Susceptibility	The haplotype of all 4 SNPs showed significant association with the disease (*p*= 0.00079). The C-A-A-TT haplotype was observed more frequently in the cases than in the controls (OR: 3.96; 95% CI: 1.90–8.21).	12
**G-611A**	Pulmonary TB	Iran and Afeghanistan	No association	There was no association between the G-611A SNP and Pulmonary TB in this study.	13
**rs1327474 (–611 A/G)**, rs11914 (+189 T/G), rs7749390 (+95 C/T), and rs137854905 (27bp ins/del)	Pulmonary TB	Iran	No association with the -611 A/G SNP	rs11914 TG genotypes decreased the risk of Pulmonary TB in comparison with TT (OR = 0.36, 95% CI: 0.21–0.62; *p*= 0.0002). The rs11914 G allele decreased the risk compared with T allele (OR= 0.41; 95% CI: 0.25–0.68; *p*= 0.0006). *IFNGR1* rs7749390 CT genotype decreased the risk in comparison with CC genotype (OR= 0.55; 95% CI: 0.32–0.95; *p*= 0.038).	14
**rs1327474 (A/G)** and other 10 SNPs	Pulmonary TB	Korea	No association with the rs1327474 (A/G)	There was no association between the rs1327474 SNP and Pulmonary TB in this study.	15
**G-611A**	Pulmonary TB	Iran	Susceptibility	There was a significant difference between the TB patients and controls for –611 genotypes and alleles of IFN-γR1 (*p*= 0.01 for both).	16
**-611 T/C** and -56 A/G	Brucellosis	Iran	Susceptibility	The genotype frequency of -611 CC was significantly higher in patients than controls (*p* <0.024; OR= 4; 95% CI: 1.21-13.3).	17

Abbreviations: OR: Odds Ratio; 95% CI: 95% Confidence Interval; TB: Tuberculosis; *M. tuberculosis*: *Mycobacterium tuberculosis*.

References

1. Koch O, Awomoyi A, Usen S, Jallow M, Richardson A, Hull J, et al. *IFNGR1* gene promoter polymorphisms and susceptibility to cerebral malaria. J Infect Dis (2002) 185:1684–7. doi:10.1086/340516

2. Awomoyi AA, Nejentsev S, Richardson A, Hull J, Koch O, Podinovskaia M, et al. No association between interferon-gamma receptor-1 gene polymorphism and pulmonary tuberculosis in a Gambian population sample. Thorax (2004) 59:291-4. doi:10.1136/thx.2003.013029

3. Rosenzweig SD, Schäffer AA, Ding L, Sullivan R, Enyedi B, Yim J-J, et al. Interferon-gamma receptor 1 promoter polymorphisms: population distribution and functional implications. Clin Immunol (2004) 112:113-9. doi:10.1016/j.clim.2004.03.018
4. Blanton RE, Salam EA, Ehsan A, King CH, Goddard KA. Schistosomal hepatic fibrosis and the interferon gamma receptor: a linkage analysis using single-nucleotide polymorphic markers. Eur J Hum Genet (2005) 13:660–8. doi:10.1038/sj.ejhg.5201388
5. Bulat-Kardum L, Etokebe GE, Knezevic J, Balen S, Matakovic-Mileusnic N, Zaputovic L, et al. Interferon-gamma receptor-1 gene promoter polymorphisms (G-611A; T-56C) and susceptibility to tuberculosis. Scand J Immunol (2006) 63:142–50. doi:10.1111/j.1365-3083.2005.01694.x
6. Falleti E, Fabris C, Toniutto P, Fontanini E, Cussigh A, Caldato M, Rossi E, et al. Cytokines and liver fibrosis progression due to recurrent hepatitis C. J Interf Cytok Res (2007) 27:239-46. doi:10.1089/jir.2006.0062
7. Hwang JH, Kim EJ, Kim SY, Lee S-H, Suh GY, Kwon OJ, et al. Polymorphisms of interferon-gamma and interferon-gamma receptor 1 genes and pulmonary tuberculosis in Koreans. Respirology (2007) 12:906-10. doi:10.1111/j.1440-1843.2007.01171.x
8. Hwang JH, Kim EJ, Koh W-J, Kim SY, Lee S-H, Suh GY, et al. Polymorphisms of interferon-gamma and interferon-gamma receptor 1 genes and non-tuberculous mycobacterial lung diseases. Tuberculosis (Edinb) (2007) 87:166-71. doi:10.1016/j.tube.2006.07.003
9. Stein CM, Zalwango S, Chiunda AB, Millard C, Leontiev DV, Horvath AL, et al. Linkage and association analysis of candidate genes for TB and TNFα cytokine expression: evidence for association with IFNGR1, IL-10, and TNF receptor 1 genes. Hum Genet (2007) 121:663–73. doi:10.1007/s00439-007-0357-8
10. Velez DR, Hulme WF, Myers JL, Weinberg JB, Levesque MC, Stryjewski ME, et al. *NOS2A*, *TLR4*, and *IFNGR1* interactions influence pulmonary tuberculosis susceptibility in African-Americans. Hum Genet (2009) 126:643–53. doi:10.1007/s00439-009-0713-y
11. Zhou J, Chen DQ, Poon VK, Zeng Y, Ng F, Lu L, et al. A regulatory polymorphism in interferon-gamma receptor 1 promoter is associated with the susceptibility to chronic hepatitis B virus infection. Immunogenetics (2009) 61:423-30. doi: 10.1007/s00251-009-0377-8
12. He J, Wang J, Lei D, Ding S. Analysis of functional SNP in *Ifng*/*Ifngr1* in Chinese Han population with tuberculosis. Scand J Immunol (2010) 71:452–8. doi:10.1111/j.1365-3083.2010.02393.x
13. Varahram M, Farnia P, Nasiri MJ, Karahrudi MA, Dizagie MK, Velayati AA. Association of *Mycobacterium tuberculosis* lineages with IFN-γ and TNF-α gene polymorphisms among pulmonary tuberculosis patients. Mediterr J Hematol Infect Dis (2014) 6:e2014015. doi:10.4084/MJHID.2014.015
14. Naderi M, Hashemi M, Rezaei M, Safdari A. Association of Genetic Polymorphisms of *IFNGR1* with the Risk of Pulmonary Tuberculosis in Zahedan, Southeast Iran. Tuberc Res Treat (2015) 2015:292505. doi: 10.1155/2015/292505
15. Shin J-G, Park BL, Kim LH, Namgoong S, Kim JO, Chang HS, et al. Association study of polymorphisms in interferon-γ receptor genes with the risk of pulmonary tuberculosis. Mol Med Rep (2015) 12:1568-78. doi:10.3892/mmr.2015.3544
16. Shamsi M, Zolfaghari MR, Farnia P. Association of IFN-γ and P2X7 Receptor Gene Polymorphisms in Susceptibility to Tuberculosis Among Iranian Patients. Acta Microbiol Immunol Hung (2016) 63:93-101. doi:10.1556/030.63.2016.1.7
17. Naseri Z, Bahmani N, Alikhani MY, Hashemi SH, Roshanaei G. Polymorphisms in Promoter Region of the Interferon-Gamma Receptor-1 Gene and its Relation with Susceptibility to Brucellosis. Iran J Pathol (2019) 14:206–11. doi:10.30699/ijp.2019.91536.1888
